# Population Ageing in Ghana: Research Gaps and the Way Forward

**DOI:** 10.4061/2010/672157

**Published:** 2010-09-29

**Authors:** Chuks J. Mba

**Affiliations:** Regional Institute for Population Studies, University of Ghana, P.O. Box 96, Legon, Ghana

## Abstract

This paper attempts to highlight research gaps and what should be done concerning population ageing in the Ghanaian context. The proportion of the elderly increased from 4.9 percent in 1960 to 7.2 percent in 2000, while the number rose from 0.3 million to 1.4 million over the same period (an increase of 367 percent). Projection results indicate that by 2050, the aged population will account for 14.1 percent of the total population. Very little is known about the living arrangements and health profile of Ghana's older population. With increasing urbanization and modernization, it is important to know something about intergenerational transfers from adult children to their elderly parents, and characterize the elderly persons' food security strategies. Training of researchers will be important in terms of strengthening Ghana's capacity to monitor trends, as well as to conduct research and explore new directions in population ageing research.

## 1. Introduction

The population aged 60 years or over tripled from its number in 1950 to 600 million in 2000, and by 2006, the number of older persons had surpassed 700 million, while current projections suggest that by 2050, 2 billion older persons will be alive, implying that their number will once again triple over a span of 50 years [1]. 

The numerical growth of elderly persons (population aged 60+ years) around the world is an eloquent testimony not only of reductions in fertility but also of reductions in infant and maternal mortality, improved nutrition, reduction in infectious and parasitic diseases, as well as improvement in health care, education, and income. Global total fertility rate has declined from 5.0 live births per woman in 1950–1955 to 2.7 live births per woman in 2000–2005, and is expected to further reduce to replacement level, that is 2.2 live births per woman by 2045–2050 period [1–3]. Also life expectancy has increased from 46.5 years in 1950–1955 to 66.0 years in 2000–2005, and is expected to rise to 76 years by the 2045–2050. In sub-Saharan Africa, the corresponding fertility values are 6.7 live births per woman in the early 1950s to 5.5 live births per woman by early 2000s and 2.4 live births per woman by 2045–2050 period. Similarly, expectation of life at birth rose from 36.7 years in the 1950s to 48.4 years by 2000–2005, and is projected to peak at 68.4 years during the 2045–2050 period. Lesotho's fertility and mortality profile is similar to that of sub-Saharan Africa. This is because fertility fell from 5.8 to 4.5 live births per woman in one half century, and is expected to fall to replacement level during 2045–2050 period, while life expectancy increased from 38.5 to 40.2 years in five decades, and is expected to reach 65.1 years by 2045–2050 period [1, 3]. 

Empirical evidence indicates that most nations will face population ageing to some degree over the next decades (see, e.g., [1–9] (Mba, 2001)), and planning for this ageing can mitigate some of the negative effects and enhance the positive consequences.

The age structure of a population is the result of the three basic population processes: fertility, mortality, and migration. When these processes are constant for many years, a stable age structure emerges [10]. Changes in fertility, mortality, or migration will produce as long-term effects. For example, the postwar baby boom of the United States initially made the population age structure younger. As the members of the baby boom cohort age, the U.S. population also ages, even if there are no future changes in fertility, mortality, or migration, due to the momentum inherent in the existing age structure and vital rates. Age structures, therefore, reflect current patterns of fertility, mortality, and migration, as well as the effects of these processes in the past [10].

In Ghana, and for most countries, fertility and mortality affect the age structure more than migration does, but at the town, city, or regional level, migration may become more important. (A classic example of this in Ghana is the repopulation and re-emergence of economic activity in the once deserted onchocerciasis-infested areas, which has now been freed of the debilitating and disabling disease [11], (Government of Ghana, 1991]. The age structure of those areas were significantly different from others during the onchocerciasis infestation due to massive out-migration). The age structure of a country, in contrast, depends more on fertility and mortality. High fertility and high mortality ultimately produce a young population whereas low fertility and low mortality in the long run produce an older population.

In Ghana, as in all parts of the world, the demographic transition is under way, although the pace and stage of the transition varies. (The demographic transition refers to the predictable shift from high mortality and high fertility to low mortality and low fertility (for more complete explanations see, e.g., (Mba, 2002); (Myers, 1990); [10]). As countries progress through the stages of demographic transition, changes in population structure, notably size and age composition, so that by the end of the demographic transition, a typical population is both larger and older.). In Ghana, mortality is still moderately high as life expectancy at birth of is still below 60 years and fertility (measured by total fertility rate) has declined moderately from 6.4 in 1988 to 4.4 children per woman in 2003 and then to 4.0 in 2008 [1, 2, 12–14]. For Ghana, the age structure is growing younger because mortality declines tend to occur first at the younger ages, as more babies survive into childhood. This initial “younging” of the population provides momentum for large population growth when these children themselves reach childbearing ages. Reducing fertility rates, along with continued efforts to sustain the mortality declines has been a primary concern of the government of Ghana as highlighted in the revised population policy document [15]. However, what is not recognized is that sustained efforts designed to reduce fertility and mortality have important implications for other aspects of the age structure of the Ghanaian population, particularly the elderly subgroup.

Although Ghana can still be classified as a youthful population [1, 2]; (Ghana Statistical Service, 2005), reductions in fertility and mortality have resulted in increase in both the proportion and absolute number of elderly population (persons aged 60 years and over) as evidenced by census results [16] and estimates from other sources [1, 2, 17]. In fact, with the proportion of the elderly population currently at 7.2 percent, Ghana has one of the highest proportions of persons aged 60+ years in sub-Saharan Africa. With continued campaigns to reduce fertility and mortality, it expected that this proportion will rise in the coming decades. What is not known is the preparedness of the country for significant changes in its age structure in response to this demographic transition.

Consequently, this paper attempts to make a modest contribution toward raising awareness regarding the phenomenon of population ageing and research gaps in the Ghanaian context. The paper is broadly segmented into two parts, namely, using empirical evidence to characterize Ghana's past, present, and future population structure; and secondly, discussing the research gaps and what should be done.

## 2. Methodology

The study utilizes the 1960–2000 census results of Ghana, and the United Nations [18] medium variant projection assumptions. The medium variant projections are used because of their direct relevance to policy formulation and decision making. In order to provide a veritable platform for raising awareness on ageing as a policy and research issue in Ghana, population projections by the component method are carried out using the SPECTRUM software developed by The Futures Group International [19]. Since the most recent census in Ghana was conducted in 2000, the base year for the projection is 2000. In the projection assumption, it is hypothesized that fertility will decline from the current level of 3.99 children per woman in such a way that Ghana will attain replacement level fertility (i.e., 2.1 children per woman) by 2050 [12, 16, 18]. Mortality is projected on the basis of the models of change of life expectancy produced by the United Nations [18]. Currently, the median prevalence rate of human immunodeficiency virus (HIV), the virus that causes acquired immunodeficiency syndrome (AIDS), is 2.7 percent of the adult population in Ghana [20]. Thus, the mortality assumption incorporates minor future impact of the HIV/AIDS epidemic based on empirical information from the country. A single migration assumption that sets international migration equal to zero, has been incorporated based on an extrapolation of past international migration estimates [16]. The basic demographic assumptions used as inputs for the projection analysis are summarized in Table 7 in the appendix. However, further details of the component projection method employed here and the underlying assumptions are presented elsewhere [21].

In depicting the economic consequences of population ageing, the median, aged dependency ratio, extreme aged, total dependency ratio, and family support ratio will be employed. The median population age, that shows the age above and below which there are equal numbers of persons, is one of the conventional indices used for assessing the process of population aging [22]. One consequence of population aging is a change in the balance of those who potentially need economic and physical support, and those who can provide such support. A rough statistical indication of this change is approximated by the overall or total dependency ratio, represented by children under age 15 plus persons aged 60 and over (number of dependent persons) per 100 persons aged 15–59 years (productive ages). This age boundary is appropriate because the official mandatory retirement age in Ghana is 60 years. The aged dependency ratio derives from the total dependency ratio and is simply persons aged 60 and over per 100 persons aged 15–59 years. It should be stated that the dependency ratio does not capture the dependents well because in the Ghanaian context, and indeed as obtains in many African countries, not all persons aged 60 and over are economically dependent; similarly, not all persons aged 15–59 years are economically independent, especially given the high unemployment rate in the face of serious economic predicament in the country [23]. In this study, two more similarly computed measures are added. The first is the extreme aged dependency ratio, which is simply the number of persons aged 80 and over per 100 persons of productive ages. The second is the family support ratio, which is the number of persons aged 80 and over per 100 persons aged 60–69 years. The family support ratio is predicated on the assumption that the “young” old children of the “oldest” old are likely to be the primary caregivers for the very old generation and that this care is generally provided within the context of the family [8].

## 3. Results

### 3.1. Trends in Age and Sex Structure

The dynamics of a country's age-sex structure are pivotal to most demographic, health, social, and economic studies. In particular, the demographic profile of a country is incomplete without an examination of the country's age and sex distribution. Knowledge of the proportion of persons in the conventional five-year age groups and broad age groups has implications for planning in health, education, and social services. The age and sex structure of Ghana's population for the period 1960–2000 is presented in Table 1. The age and sex distribution reveals that there are more persons in the younger age groups due to past high fertility and concomitant rapid population growth. However, because of the impact of mortality, which increases with age, the proportion of persons decreases with advancing age. 

The table shows that children below 15 years of age have consistently constituted the largest proportion of the country's population over the years. About 4 in every 10 Ghanaians are children aged 0–14 years, implying that Ghana is still a young population, a situation that is characteristic of a developing country. However, the proportion of these children has been declining since 1984 as a result of declining fertility and uptake in female formal education [16].

The proportion of persons aged 15–60 years has generally remained around 50 percent over the years, while persons aged 60 years and above have the least proportion in the population. What can be said of both sexes applies also to either sex. But whereas there were more males than females in 1960, the sex ratio (the proportion of males per 100 females) shifted in favour of females in subsequent censuses. 

It is interesting to note that although Ghana's age-sex structure has not changed much during the period under review, both the number and proportion of persons aged 60 years and over have been increasing.

### 3.2. Ageing Trends

Table 2 depicts the percentage distribution of Ghana's elderly population, that is, persons aged 60 years and over, by sex for the period 1960–2000. As should be expected, there are more people aged 60–64 years than in the succeeding age groups since they are younger and *ceteris paribus *the force of mortality is felt much more in the older than younger ages. This pattern is maintained for both sexes and each sex. What is striking about the table is that the proportion of the aged population in each age group has generally risen over the years. Additionally, both the number and proportion of the elderly to the total population have consistently for both sexes and for each sex. The proportion of the elderly to the total population increased from 4.9 percent in 1960 to 7.2 percent in 2000, while the number rose from 0.3 million to 1.4 million over the same period (an increase of 367 percent). The increase in the number and proportion of the elderly persons lends itself to a number of factors. Paramount among these are improvements in life expectancy (resulting in more people surviving to old age) precipitated by improved public health measures, better nutrition and personal hygiene, and declining fertility, which reduces the share of the young children to the total population.

Evidence from many countries suggests that women live longer than men (see, e.g., [1–3, 9, 18, 24]). Also, because women marry men much older than themselves, especially in parts of Africa, they are expected to survive their husbands. As a result, more elderly women than men should be expected at older ages. However, Table 2 does not tend to support this hypothesis as there seems to be more older men than women from the census results. Nevertheless, the table seems to suggest that the phenomenon of ageing is emerging in Ghana's population.

Table 3 reveals that there are consistently more elderly persons in those regions more suitable for agricultural production (Western, Eastern, Ashanti, and Brong Ahafo Regions), suggesting that these regions attract more migrant farmers than the other regions. It is striking to note that Greater Accra Region, Ghana's capital, which is the most urbanized (urbanization is the process of population concentration in towns, cities, and metropolitan areas, or simply an increase in the proportion of a population residing in urban areas) region in the country [12, 14] is among the regions with the least proportion of elderly persons, supporting the argument that people who live in towns and cities return to their villages as they grow old to, among other things, find sustenance in agricultural production. The table also reinforces the previous discussion that Ghana's elderly population is concentrated in the younger ages (60–69 years), and that the proportion of the elderly people to the total population has risen appreciably, especially in 2000. Consequently, it can be argued that internal migration in Ghana may be a rational response to the wide disparities in economic and social conditions between the rural and urban areas. Additionally, many migrants return to rural communities when they grow old since they may have retired from employment and can no longer cope with the high cost of living in towns and cities. For example, it has been found among the Ashantis that the need to return home becomes increasingly important as they age because the men and women become eligible for leadership roles as lineage elders and household heads [11, 25]. (The Ashantis predominate in the Ashanti Region of Ghana, and live in the heart of the cocoa growing zone in central Ghana.) 

Table 4 highlights gender disparities with respect to selected socioeconomic variables concerning the phenomenon of population ageing in Ghana between 1984 and 2000. The table reveals that an overwhelming majority of older men and women reside in rural Ghana. More elderly women than men are likely to be found in both villages and towns as the sex ratios, suggesting a deficit of males, especially in 2000. Most of the elderly men and women have not had any formal education, although there is a slight improvement between 1984 and 2000, particularly with respect to older men. The evidence further indicates that about four out of every five older men or women are engaged in agricultural activities. 

Table 4 further suggests that while majority of the older men are likely to be married, majority of their female counterparts are likely to be widowed/divorced/separated. In all, while there are 98 males for every 100 females in the entire population [12, 14], there are only 87 older men for every 100 older women for the elderly population, as shown in the last column of Table 4. The disparities with respect to these characteristics may be due to the fact that women, on average, live longer than men in most populations [1–3, 9, 18] and typically marry men older than themselves, and men are more likely than women to remarry after divorce or the death of a spouse [26]. Additionally, since many young adults, especially males, are more likely to migrate to cities in search of work and schools, some of them may choose to remain in the cities where social amenities and other benefits of modernization are concentrated after retirement, while others return to their villages to subsist on agriculture due to inadequate or lack of financial base to cope with the demands of city life [4, 5, 7]. (It should be noted that social attachments of the migrants with their childhood areas may also induce some of them to return to the rural areas after retirement.)

Although the percentage of the elderly persons has risen significantly in rural Ghana, there is no evidence to suggest a corresponding social care for the aged. In spite of the demographic shift, older persons' concerns have remained marginal to the major social and economic debates in the country. As a result, many older people, who cannot engage in large-scale agricultural activities, are faced with inadequate and insecure income in the absence of extended family support. 

Table 5 depicts Ghana's future age structure. The table suggests that while children under 15 years will constitute about 36.9 percent of the total population, and persons aged 60 years and over accounting for 6.1 percent of the total population in 2010, by the year 2030, children under 15 years and persons aged 60 years and over will account for about 29.4 percent and 8.6 percent, respectively, of the total population. Then by 2050, it is expected that the proportion of children under 15 years will further reduce to 22.3 percent while the aged population will account for 14.1 percent of the total population. In absolute numbers, persons aged 60 years and over will increase from 1.5 million in 2010 to 2.8 million in 2030, and then to 5.7 million. Similarly, the oldest old, persons aged 80+ years, will consistently rise over the projection period, peaking at 1.4 percent of the total population (or 0.6 million) in 2050. 

The results of indicators of population ageing are presented in Table 6. Ghana's median age is envisaged to rise to 23.5 years in 2020, representing an *intermediate* population, and to 31.7 years by 2050, representing an *old* population. It is projected that the overall dependency ratio will decline to 57.2 percent by 2050, while the aged dependency ratio will rise to 22.2 percent by 2050. Thus the coming decades will witness remarkable shifts in Ghana's population age structure toward older ages as a result of decline in the proportion of children under 15 years. The last two rows of Table 4 reveal that the envisaged shifts in Ghana's age structure toward the elderly will be concentrated especially among the young old, that is, those aged 60–69 years because the extreme aged dependency ratio has changed only minutely during the projection period, while the family support ratio declined.

## 4. Discussion, Research Gaps, and the Way Forward

From the preceding discussion, several critical issues relating to population aging research in Ghana have emerged. Research which will inform policy and planning for an aging population is a priority area in the country. 

As a result of the anticipated shifts in Ghana's population age structure toward older age groups precipitated by its demographic transition process, the government should utilize available time to prepare for a future aging society by formulating effective policies and programs before population ageing becomes a substantial public and private burden. 

The preceding analysis has shown that the elderly will be concentrated more in the young age groups, that is, 60–69 age range. Thus, most of Ghana's older population will consist of individuals in the younger part of that age group, probably not yet in need of extensive health services and able to continue working in some capacity. Ghana's older population instead could become a resource for economic development [27]. 

It is conceded that the foregoing population projection hypotheses may be imprecise because population projections are as good as the assumptions underlying them so that different assumptions will result in different population figures. Given the unpredictability of both human and reproductive behaviour, these estimates should be accepted with caution. However, the results are suggestive of what might happen in future. In particular, the population projections are needful in order to provide a veritable platform for raising awareness on aging as a policy and research issue in Ghana. 

The results of the study have shown the preponderance of older women relative to their male counterparts. Beside taking care of their grandchildren, meals, housework, and taking part in community affairs, elderly women in Ghana engage in much agricultural work that includes planting, weeding, watering, harvesting, processing, and storage of the food their families eat. In fact, it has been found that in most activities in the rural areas, the roles of older wives are not statistically different from their younger counterparts [28]. Elderly women farmers often make long and tedious journeys to market, sometimes with grandchildren on their backs and carrying heavy headloads of farm produce to sell. Additionally, in many rural areas, the aged women are the heads of the households mainly because of the death of their husbands. Despite the fact that they do much agricultural work, the rural elderly women are yet to benefit from agricultural support programmes such as extension and credit. Due to reasons that include lack of adequate sensitization of the power brokers through research and dissemination of research findings (that should highlight older persons' domestic duties, status, false assumptions, and illiteracy) men still receive most loans and control most of the land, while most of Ghana's real farmers are women. In fact, according to the World Bank [29], women in Ghana constitute 47 percent of total labour force in agriculture, and they account for as much as 70 percent of the total food production. Consequently, research efforts that will furnish an understanding of women farmers' role and its importance, as well as these constraints are a prerequisite to devising policies to improve productivity and socioeconomic development.

The ageing process exposes individuals to increasing risk of illness and disability. As Ghana is a poor country, lifetime exposure to health problems means that many Ghanaians may enter old age already in chronic ill-health. Personal health consistently ranks alongside material security as a priority concern for the aged. Indeed, physical health is for many rural elderly persons their single most important asset, bound up with their ability to work in the farms, to function independently, and to maintain a reasonable standard of living. But very little is known about the health profile of Ghana's older population.

Due to pervasive poverty, it is hypothesized that there is an inverse relationship between modernization and family support for the elderly, resulting in a growing incidence of low levels of well-being among the elderly persons. Yet, very little is known in Ghana and other parts of Africa about intergenerational transfers. In the traditional African society, children are expected to support their parents in old age because there is no universal social security system. With increasing urbanization and modernization, it is vitally important to know something about intergenerational transfers from adult children (who live in towns, cities, and outside the country) to their elderly parents, and characterize the elderly persons' food security strategies in a fast-changing social and economic environment.

Unfortunately, Ghana and many countries in Africa have accorded relatively low priority in their national policies to the ageing of their populations. As a result of lack of full knowledge of the implications of the changes taking place in the traditional family, it is still assumed in most of these settings that the family will continue to provide the context within which the needs of the older population could be met.

Training of researchers will be important in terms of strengthening Ghana's capacity to monitor trends, as well as to conduct research and explore new directions in population ageing research. Also, subregional networks that could facilitate exchange of information, resource sharing, training opportunities, and, more importantly, effective dissemination of research findings are increasingly needful. Such networks may be developed by the relevant international agencies in conjunction with appropriate persons at the national level to ensure optimal cooperation and success of research endeavors.

Although old people have existed throughout recorded history, few studies have been done to systematically investigate attitudes toward old age and the elderly persons' actual conditions in Ghana and many African countries. Such research is absolutely essential because we need to have reliable baseline information by which to assess the current conditions of the aged and on which to premise our projections and expectations about old people's situation in the future. 

The pace of population ageing is faster in developing countries than in developed countries. Consequently, developing countries will have less time to adjust to the consequences of population ageing. Moreover, population ageing in developing countries is taking place at lower levels of socioeconomic development than has been the case for developed countries.

Furthermore, population ageing is profound, having major consequences and implications for all facets of human life. In the economic area, population ageing will have an impact on economic growth, savings, investment, consumption, labour markets, pensions, taxation, and intergenerational transfers. In the social sphere, population ageing influences family composition and living arrangements, housing demand, migration trends, epidemiology, and the need for healthcare services. In the political arena, population ageing may shape voting patterns and political representation.

The foregoing presentation points to the fact that ageing should be one of the most important issues that need to be addressed in the country. In the developed countries, the demographic transition process leading to an ageing population has taken place over the span of more than a century [30–32]. This furnished plenty of warning and preparation time for increased numbers of elderly people. In Ghana in particular and Africa in general, this process of transition has occurred in a few decades. It should be noted that even countries with a high level of economic and social development would find it difficult adjusting to a rapidly ageing population in such a short time period. Then for countries still struggling with the problems of underdevelopment, where unfortunately most of these African countries are currently located, the challenges will be undeniably formidable. The government of Ghana, and indeed African governments, should be aware of this “time bomb” before the situation gets out of hand because there is almost no social security benefits for most of the aged in much of Africa, majority of whom are illiterates and who therefore could not enjoy the benefits of formal employment and the concomitant pensions. 

Because of these foregoing reasons, it is important that our developing country governments are sufficiently sensitized. In particular, the government of Ghana should know about the consequences and implications of the phenomenon of population ageing in order to plan for the “rainy day.” One way of doing this is to always highlight the challenges precipitated by population ageing in the country, detailing research gaps and proffering plausible solutions.

## Figures and Tables

**Table 1:** Percentage distribution of Ghana's age-sex structure by five-year age groups: 1960–2000.

Age group	1960			1970			1984			2000		
	Both sexes	Male	Female	Both sexes	Male	Female	Both sexes	Male	Female	Both sexes	Male	Female
0–4	19.28	18.89	19.67	18.27	18.32	18.21	16.51	16.74	16.29	14.57	14.74	14.54
5–9	15.14	15.16	15.12	16.94	17.14	16.74	16.28	16.70	15.87	14.67	14.86	14.49
10–14	10.13	10.52	9.72	11.71	12.11	11.32	12.23	12.78	11.69	11.96	12.30	11.63
15–19	8.04	8.10	7.98	9.09	9.39	8.79	10.14	10.50	9.78	9.96	10.27	9.66
20–24	8.78	7.89	9.70	7.96	7.19	8.71	8.59	7.98	9.18	8.46	8.15	8.77
25–29	8.70	8.19	9.21	7.38	6.83	7.92	7.69	7.15	8.21	7.86	7.43	8.29
30–34	7.26	7.13	7.39	6.55	6.21	6.89	6.04	5.80	6.28	6.38	6.05	6.70
35–39	5.61	5.83	5.39	5.12	5.21	5.03	4.75	4.66	4.84	5.45	5.25	5.64
40–44	4.63	4.88	4.38	4.09	4.11	4.07	3.85	3.73	3.97	4.69	4.74	4.64
45–49	3.25	3.61	2.87	3.18	3.39	2.97	3.48	3.58	3.39	3.81	4.03	3.59
50–54	2.65	2.85	2.46	2.70	2.82	2.59	2.87	2.86	2.88	3.01	2.99	3.02
55–59	1.60	1.74	1.46	1.67	1.80	1.53	1.73	1.77	1.70	1.88	1.95	1.81
60–64	1.75	1.87	1.46	1.71	1.77	1.53	1.84	1.78	1.89	1.94	1.90	1.98
65–69	0.91	0.95	0.86	1.10	1.12	1.06	1.18	1.16	1.20	1.37	1.38	1.36
70–74	0.84	0.88	0.80	0.96	0.99	0.94	1.05	1.05	1.04	1.19	1.14	1.24
75–79	0.46	0.48	0.44	0.49	0.51	0.48	0.58	0.60	0.37	0.77	0.79	0.74
80+	0.96	1.02	0.90	1.08	1.09	1.08	1.20	1.17	1.23	1.96	2.01	1.91

Total	100.00	100.00	100.00	100.00	100.00	100.00	100.00	100.00	100.00	100.00	100.00	100.00

Number	6,726, 815	3,399, 908	3,326, 907	8,559, 313	4,212, 883	4,349, 430	12,296, 081	6,063, 526	6,232, 555	18,912, 079	9,357, 382	9,554, 697

Sources: The 1960, 1970, 1984, and 2000 Population Censuses of Ghana.

**Table 2:** Percentage distribution of Ghana's elderly population by sex: 1960–2000.

Age group	1960			1970			1984			2000		
	Both Sexes	Male	Female	Both Sexes	Male	Female	Both Sexes	Male	Female	Both Sexes	Male	Female
60–64	1.75	1.87	1.46	1.71	1.77	1.53	1.84	1.78	1.89	1.94	1.90	1.98
65–69	0.91	0.95	0.86	1.10	1.12	1.06	1.18	1.16	1.20	1.37	1.38	1.36
70–74	0.84	0.88	0.80	0.96	0.99	0.94	1.05	1.05	1.04	1.19	1.14	1.24
75–79	0.46	0.48	0.44	0.49	0.51	0.48	0.58	0.60	0.37	0.77	0.79	0.74
80+	0.96	1.02	0.90	1.08	1.09	1.08	1.20	1.17	1.23	1.96	2.01	1.91

Total	4.92	5.20	4.46	5.34	5.48	5.09	5.85	5.76	5.73	7.23	7.22	7.23

Number	325,178	176,814	148,364	452,236	232,780	219,456	706,385	349,278	357,107	1,366,408	675,603	690,805

Sources: the 1960, 1970, 1984, and 2000 population censuses of ghana.

Note. The percentages refer to proportions of the aged population to the total country population.

**Table 3:** Proportion of Ghana's elderly to total population by region, 1960–2000.

Region	Percentage											
	60–69 years				70–79 years				80+ years			
	1960	1970	1984	2000	1960	1970	1984	2000	1960	1970	1984	2000
All regions	2.2	2.2	2.4	3.8	1.1	1.2	1.2	1.9	0.8	1.0	1.0	2.2
Western	2.1*	2.3	2.5	3.8	1.3*	1.3	1.4	1.9	0.9*	0.9	1.1	2.6
Central	—	1.9	2.3	3.9	—	1.2	1.2	1.7	—	1.2	1.2	2.1
Greater Accra	2.2	2.3	2.2	3.0	1.0	1.1	1.2	1.6	0.6	0.6	0.7	1.8
Volta	1.8	1.9	1.9	3.4	1.3	1.3	1.3	1.9	0.9	1.1	1.3	2.7
Eastern	1.9	2.0	2.3	3.9	1.0	1.2	1.2	2.0	0.8	1.3	1.3	2.4
Ashanti	2.3	2.3	2.4	4.1	1.1	1.2	1.2	1.8	0.9	1.2	1.1	2.7
Brong Ahafo	2.3	2.2	2.4	3.9	0.9	1.3	1.3	2.1	0.8	1.2	1.2	2.4
Northern	2.2^+^	2.1	2.1	3.6	1.1^+^	1.2	1.1	1.8	0.9^+^	0.9	1.0	2.0
Upper East	—	—	1.9	3.7	—	—	1.2	1.8	—	—	0.9	2.0
Upper West	—	2.2^∧^	2.3	3.7	—	1.1^∧^	1.2	1.9	—	1.1^∧^	0.9	1.9

Sources: 1960–2000 Ghana population censuses.

Note. *Includes the present central region.

^+^Includes the present Upper East and Upper West regions.

^∧^Includes the present Upper East region.

**Table 4:** Percentage distribution of elderly persons aged 60 and above in Ghana by selected characteristics, 1984–2000.

Characteristics	1984			2000			Sex ratio	
	Male	Female	Total	Male	Female	Total	1984	2000
*Place of residence*								
Rural	77.4	72.1	74.7	61.7	60.7	61.2	103.7	88.4
Urban	22.6	27.9	25.3	38.3	39.3	38.8	78.1	85.0

*Education*								
No education	71.1	77.7	74.8	60.8	70.2	66.6	91.1	88.9
Primary	18.9	20.0	19.3	23.7	21.5	22.2	93.4	102.1
Secondary	5.7	2.1	3.6	9.3	5.2	7.1	112.2	117.8
Tertiary	4.3	0.2	2.3	6.2	3.1	4.1	127.6	130.4

*Occupational status*								
Agricultural	78.2	86.5	83.7	74.9	82.7	78.9	87.6	86.9
Nonagricultural	21.8	13.5	16.3	25.1	17.3	21.1	109.9	107.8

*Marital status*								
Never married	4.4	9.2	7.7	3.6	8.8	5.4	82.7	80.9
Married	59.5	40.6	47.9	62.6	42.3	50.9	117.9	129.9
Widowed/divorced/separated	36.1	50.2	44.4	33.8	48.9	43.7	77.7	80.1
Number	349263	361872	711135	631395	725280	1356675	96.5	87.1

Sources: 1984 and 2000 censuses of Ghana.

**Table 5:** Future structure of Ghana's population, 2010–2050.

Year	Number (thousands)				Percent distribution			
	0–14	15–59	60+	80+	0–14	15–60	60+	80+
2010	8971	13858	1483	122	36.9	57.0	6.1	0.5
2015	9350	15486	1727	159	35.2	58.3	6.5	0.6
2020	9587	17158	2044	202	33.3	59.6	7.1	0.7
2025	9568	18981	2415	248	30.9	61.3	7.8	0.8
2030	9724	20507	2844	298	29.4	62.0	8.6	0.9
2035	9797	22017	3301	351	27.9	62.7	9.4	1.0
2040	9785	23351	3929	408	26.4	63.0	10.6	1.1
2045	9685	24621	4590	506	24.9	63.3	11.8	1.3
2050	9048	25804	5721	568	22.3	63.6	14.1	1.4

**Table 6:** Measures of population aging in Ghana: 2010–2050.

Measure	Year				
	2010	2020	2030	2040	*2050*
Median	21.0	23.5	26.0	28.8	*31.7*
overall dependency ratio	75.4	67.8	61.3	58.7	*57.2*
Aged dependency ratio	10.7	11.9	13.9	16.8	*22.2*
Extreme aged dependency ratio	0.8	1.2	1.5	1.7	*2.2*
*Family support ratio*	*31.2*	*30.1*	*27.2*	*24.5*	*21.2*

Source: computed from Table 5.

Note. The median is expressed in years while the ratios are expressed in percent.

**Table 7:** Demographic assumptions for Ghana's population projections: 2000–2050.

Year	Total fertility	Male life	Female life	Net migration
	rate	Expectancy	Expectancy	
2000	3.99	56.0	57.7	Constant
2010	3.37	57.6	58.5	Constant
2020	3.01	61.1	62.6	Constant
2030	2.70	64.4	66.6	Constant
2040	2.44	67.2	69.9	Constant
2050	2.10	69.8	72.9	Constant

Note. The mortality inputs correspond to the pattern of mortality implied by the North Family Model Life Tables (Coale and Demeny, 1983).

The total fertility rate and life expectancy are measured in number of children per woman and years, respectively, while net migration is constant at zero.

## Appendix

See Table 7.
